# The effects of concentrate to roughage ratio in the diet on growth performance, carcass traits, and meat quality of housed yaks

**DOI:** 10.1371/journal.pone.0330834

**Published:** 2025-09-11

**Authors:** Fajie Gou, Yincang Han, Yonggang Sun, Weiqing Ding, Shenwei Jin, Yaqian Liu, Jianyu Chen

**Affiliations:** 1 Academy of Animal Science and Veterinary Medicine, Qinghai University, Xining, China; 2 Key Laboratory of Plateau Livestock Genetic Resources Protection and Innovative Utilization of Qinghai Provincial, Xining, China; Tokat Gaziosmanpaşa University: Tokat Gaziosmanpasa Universitesi, TÜRKIYE

## Abstracts

This study examined the effects of different dietary concentrate-to-forage ratios on the growth performance, carcass traits, and meat quality of housed yaks. A total of 40 yaks from the Qinghai Plateau, aged 8–9 months with similar initial body weights, were randomly assigned to four dietary groups with concentrate-to-forage ratios of 80:20 (C80), 65:35 (C65), 50:50 (C50), and 35:65 (C35). Following a 15-day adaptation period, the yaks underwent a 105-day feeding trial. The results indicated a numerical increase in final body weight with higher concentrate ratios, although the differences were not statistically significant. Yaks in the C65 group exhibited significantly greater weight gain and carcass weight than those in the C50 and C35 groups (*P* < 0.05). In LD muscle, the C80 group showed significantly higher drip loss compared to the C65 and C35 groups (*P* < 0.05). Furthermore, iron content was significantly lower in the C50 compared to the other groups, whereas C20:3n6 content was lower in the C50 and C35 groups compared to the C65 and C80 groups (**P* *< 0.05). In summary, a concentrate-to-forage ratio of 65:35 was optimal for improving yak growth performance, carcass characteristics, and overall meat quality.

## Introduction

The yak (*Bos grunniens*) is a key ruminant species in the Qinghai-Tibet Plateau (QTP) of China, playing a crucial role in the livelihood of local herders [1]. Traditional yak husbandry is constrained by the high-altitude environment, where the predominant grazing system, often described as “relying on the sky to eat,” fails to meet the animals’ nutritional requirements. This results in a cyclical pattern of seasonal weight fluctuation—adequate nutrition in summer and autumn followed by severe weight loss in winter and increased mortality in spring—ultimately limiting the full growth potential of yaks. In recent years, the expansion of yak farming and the region’s harsh climatic conditions have exacerbated pasture degradation, leading to fluctuations in forage availability [2]. For instance, winter biomass in the Tibetan Plateau averages 750 kg/hm in terms of dry matter, with a crude protein content of 6.2% [3].

Under housed feeding conditions, the dietary concentrate-to-roughage ratio plays a crucial role in yak growth, carcass traits, and meat quality. In modern large-ruminant farming, high-grain diets are commonly used to maximize energy intake and support increased feed intake. Additionally, such diets promote fat deposition in meat-producing ruminants [4]. However, excessive grain feeding can lead to ruminal or metabolic acidosis, severely impairing feed conversion, gastrointestinal function, and overall animal health and welfare [5]. Conversely, a low proportion of concentrate in the diet reduces growth performance, while an insufficient roughage content increases feed intake but decreases nutrient digestion and absorption, ultimately hindering animal growth [6]. Previous studies have explored the impact of different concentrate-to-roughage ratios on ruminant performance. For instance, Jiang et al. [3] reported that a higher concentrate-to-roughage ratio (70:30) improved dry matter intake (DMI) and growth performance in yaks, decreased rumen pH, and influenced rumen epithelial morphology. Similarly, Zhang et al. [4,7] found that in Holstein heifers, higher concentrate feeding reduced dry matter (DM) and neutral detergent fiber (NDF) intake while increasing non-fiber carbohydrate (NFC) and starch consumption, which enhanced the apparent digestibility of DM, organic matter (OM), and crude protein (CP) without adverse effects. Feeding higher concentrate-to-roughage ratios (60:40 or 80:20) has been identified as a viable strategy for heifer nutrition. Optimizing the dietary concentrate-to-roughage ratio is economically significant in yak production systems for several reasons. First, it directly affects feed efficiency and growth performance, leading to shorter finishing times and reduced feeding costs per unit of weight gain. Second, improved carcass yield and meat quality can enhance the market value of yak meat, especially in high-end or export markets. Third, fine-tuning the concentrate level can help balance feed cost and animal performance, enabling producers to maximize returns under varying resource availability and economic conditions. Therefore, identifying an optimal ratio not only improves biological efficiency but also contributes to the overall profitability and sustainability of yak farming operations. Therefore, determining an optimal dietary concentrate-to-roughage ratio that balances growth performance, carcass quality, and overall health is essential for yak production. This study investigates the effects of different dietary concentrate-to-roughage ratios on yak production performance, carcass traits, and meat quality to identify an optimal feeding strategy that promotes growth while maintaining animal health. The findings will provide a theoretical basis for practical applications in yak farming.

## Materials and methods

### Test animals and experimental design

At the conclusion of the feeding trial, all yaks were transported to a licensed commercial slaughter facility located approximately 75 kilometers from the experimental site. Transportation was conducted early in the morning to minimize stress, and animals were allowed to rest for 12 hours in holding pens with free access to water but no feed. To ensure animal welfare and compliance with ethical standards, all yaks were slaughtered under the supervision of professional slaughterhouse staff in accordance with the guidelines set by the China National Standards for the Humane Slaughter of Livestock (GB/T 19477-2004) and approved by the Animal Ethics Committee of the Institutional Animal Care and Use Committee of Qinghai University, approval number 2023-QHMKY-001. Each yak was individually restrained and rendered unconscious using a captive bolt stunning device (non-penetrating type), which was applied to the frontal region of the skull. Immediate loss of consciousness was confirmed by the absence of corneal reflex, rhythmic breathing, and response to pain stimulus. Once unconscious, the animal was suspended and exsanguinated by severing the carotid arteries and jugular veins using a sharp knife.This method ensured rapid and complete bleeding, and no animals regained consciousness during the procedure. The entire process was monitored by trained personnel to ensure strict adherence to animal welfare protocols and to minimize suffering. This study was conducted from January to May 2024 at the Bianma Meron Palm Cooperative in Qilian County, Haibei Tibetan Autonomous Prefecture, Qinghai Province, China(38°45’61”N,99°54’27”E and with average altitude 3450m). Forty Qinghai Plateau-type yaks, aged 8–9 months and in good health with similar body weights (68.725 ± 18.973 kg), were selected and randomly assigned to four groups, with 10 replicates per group. The groups were fed total mixed rations (TMR) with different concentrate-to-roughage ratios: 80:20 (C80), 65:35 (C65), 50:50 (C50), and 35:65 (C35). The experiment included a 15-day adaptation period followed by a 105-day feeding trial. The basic principle behind selecting these concentrate-to-roughage ratios is to evaluate the effects of increasing dietary energy density on yak performance. Higher concentrate levels generally improve growth and feed efficiency, while higher roughage supports rumen health and reduces cost. The chosen ratios provide a systematic gradient to identify a balanced and economically viable feeding strategy. Diets were formulated in accordance with the Chinese Standard for Beef Cattle Feeding (NY/T 815-2004), with detailed composition and nutrient content presented in Table 1. The concentrate mixture was formulated identically for all groups, and only the concentrate-to-roughage ratio was adjusted. Independent nutrient analysis of the concentrate mixture was not conducted, as the focus of the study was on the total dietary composition and its effects.

**Table 1:** Composition and nutrient level of experimental feed (dry matter basis).

Ltems	Groups			
	C80	C65	C50	C35
Ingredients				
Oat hay (%)	20	35	50	65
Corn (%)	36.5	27.5	16.7	6.5
Wheat (%)	9	6.8	5.7	4.3
Wheat bran (%)	8.5	8	7.3	7
Soybean meal (%)	7.6	5.5	5	3.5
Rapeseed meal (%)	9	8.2	7.3	6.7
Cottonseed meal (%)	6.4	6	5	4
premix^1^ (%)	2	2	2	2
NaCl (%)	1	1	1	1
total	100	100	100	100
Nutrient level				
Metabolizable energy (ME/(MJ/kg)^2^)	9.9	9.35	8.4	8.1
Dry matter (%)	84.1	83.0	80.3	75.8
Crude protein (%)	13.081	13.02	13.0	13.03
Crude ash (%)	4.94	5.58	6.33	7.04
Neutral detergent fibre (%)	61.5	60.3	54.1	59.2
Acid detergent fibre (%)	37.06	37.8	34.3	29.9
Crude fat (g/kg)	15.0	22.0	25.0	14.0
Ca (mg/kg)	286.0	507.0	524.0	396.0
P (mg/kg)	115.79	261.25	248.36	168.58

1) Each kg of premix contains Co 0.20 mg, Cu 15 mg, I 0.80 mg, Fe 100 mg, Mn 25 mg, Se 0.17 mg, Zn 50 mg, VA 3000 IU, VD 350 IU, VE 40 IU. 2) The metabolic energy of nutrient level was calculated, and the rest was measured. The nutrient composition of the oat hay: CP:10%, CF:31%, ADF:39%, NDF: 63%, EE: 2.3%, ASH: 8%, Ca:0.40%, P:0.27%. Nutrient levels reflect the total mixed diets (concentrate + oat hay). A separate analysis of the concentrate mixture was not performed in this study.

### Feeding management

Before the experiment commenced, the pens were cleaned and sterilized. The yaks were then assigned to their respective groups, ear-tagged, and housed in the designated pens. They were fed twice daily at 09:00 and 17:00, with feed provided in excess to ensure leftovers after each feeding. The remaining feed was weighed the following morning before the next feeding. Throughout the experimental period, the yaks had ad libitum access to feed and water.

### Sample collection and indicator measurement

#### Measurement of growth performance.

Yaks were weighed at the beginning of the trial and before the morning feeding at the end of the experiment. Total weight gain and average daily gain (ADG) were calculated for the entire trial period. Body height, body slant length (the distance from the end of the animal’s shoulder to the end of the sciatic bone), and chest circumference were measured before and after the trial using a tape measure to assess growth and development.

#### Determination of carcass properties.

Five yaks per treatment group were randomly selected for slaughter. Prior to slaughter, all yaks were fed and watered as usual, with a 24-hour fasting period for feed and 2 hours of fasting for water. The pre-slaughter live weight, carcass weight, carcass length, carcass depth, head mass, skin mass, hoof weight, and viscera mass were measured, and the slaughter yield was calculated.Slaughter rate = carcass mass/pre-slaughter live mass x100%;

#### Assessment of meat quality.

The longest back muscle, biceps femoris, and triceps brachii of yak carcasses with different ratios of concentrate to roughage were collected and used for the determination of meat quality.

Determination of pH, meat color, drip loss, and cooking loss was referred to Determination of Livestock and Poultry Meat Quality (NY/T1333-2007) (China), and shear force was referred to Determination of Meat Tenderness Shear Force Measurement Method (NY/T1180-2006) (China).

#### Determination of conventional nutrients, trace elements, and amino and fatty acids.

At the end of the experiment, 100 g of the longest back muscle samples were collected, stored in dry ice and then transferred to a refrigerator at −80 °C, and then tested for conventional nutrients, trace elements, amino acids and fatty acids by Qingdao Zhengxin Detection and Analysis Co.

Moisture content was determined according to GB/T 5009.3-2016 (China); crude ash based on GB/T 5009.4-2016 (China); crude protein as described in GB/T 5009.5-2016 (China); crude fat content according to GB/T 5009.6-201 (China); and Ca, P, Fe, Zn content based on GB/T 5009.268-2016 (China). Meat fatty acid determination was carried out according to GB/T 5009.168-2016 (China), while amino acid determination based on GB/T 16631-2008 (China).

### Statistics and analysis of data

Preliminary recording and processing of data was carried out using Excel software and then statistically analyzed using the one-way ANOVA test in the SPSS 27.0 statistical software, and the data are expressed as mean ± standard deviation. *P < *0.05 indicates significant difference and *P < *0.01 indicates highly significant difference.

## Results and analysis

### Effect of dietary concentrate/crude ratio on yak growth performance

As shown in Table 2, final body weight exhibited an increasing trend with higher concentrate feed inclusion (*P* > 0.10). The body slant length of yaks in the C65 group was significantly greater than that in the C50 group (*P* < 0.05). Total weight gain in yaks fed the C65 diet was significantly higher than in the C50 and C35 groups by 18.29% and 19.63%, respectively (*P* < 0.05). There were no significant differences in average daily feed intake among the four groups (*P* > 0.05).

**Table 2:** Effects of dietary concentrate/crude ratios on growth performance of yaks (Bos grunniens).

Groups	C80	C65	C50	C35	*P*-Value
Starting weight (kg)	71.60 ± 4.47	73.50 ± 3.48	70.30 ± 3.05	72.20 ± 2.81	0.199
Initial height (cm)	78.60 ± 5.68	79.40 ± 2.51	79.60 ± 4.39	78.40 ± 4.51	0.965
Initial chest circumference (cm)	111.20 ± 7.09	109.80 ± 6.26	111.80 ± 1.92	115.20 ± 3.42	0.417
Initial body slant length (cm)	92.00 ± 4.12^b^	94.20 ± 3.63^ab^	99.80 ± 7.89^a^	92.40 ± 1.67^b^	0.039
Final body weight (kg)	141.40 ± 22.71	152.40 ± 11.19	122.80 ± 26.34	125.40 ± 21.03	0.129
End body height (cm)	97.80 ± 5.22	98.60 ± 3.58	96.80 ± 5.45	96.20 ± 9.73	0.936
End chest circumference (cm)	157.80 ± 7.76	151.60 ± 4.45	151.20 ± 8.16	156.00 ± 6.74	0.375
End body slant length (cm)	117.80 ± 8.41^ab^	122.00 ± 4.53^a^	110.00 ± 8.45^b^	112.80 ± 7.05^ab^	0.019
Total weight gain (kg)	69.80 ± 1.27^ab^	74.90 ± 4.61^a^	61.20 ± 3.77^b^	61.20 ± 2.28^b^	0.046
Average daily weight gain (kg)	0.58 ± 0.08	0.62 ± 0.03	0.51 ± 0.11	0.51 ± 0.07	0.119

Peer data with the same lowercase letter indicates a non-significant difference (*P* > 0.05), and a different letter indicates a significant difference (*P* < 0.05), as shown in the table below.

### Effect of dietary concentrate/crude ratio on yak carcass properties

As shown in Table 3, the carcass weight of yaks in the C65 group was significantly higher than that in the C50 and C35 groups by 23.29% and 20.94%, respectively (*P* < 0.05). Neither carcass length and carcass depth showed significant differences with increasing concentrate-to-roughage ratios (*P* > 0.05), nor did the slaughter rate (*P* > 0.05). As shown in Table 4, regarding the effects of dietary finishing ratios on organ indices and non-carcass properties, the lung mass of yaks in the C65 group was significantly higher than that in the C80, C50, and C35 groups (*P* < 0.05).

**Table 3:** Effect of dietary concentrate ratio on yak carcass properties.

Groups	C80	C65	C50	C35	*P*-Value
ante-mortem live weight (kg)	141.40 ± 22.71	152.40 ± 11.19	122.80 ± 26.34	125.40 ± 21.03	0.129
carcass weight (kg)	70.56 ± 11.15^a^	78.22 ± 5.69^a^	60.00 ± 13.63^b^	61.84 ± 11.01^b^	0.017
carcass length (cm)	113.80 ± 8.95	115.60 ± 3.36	102.60 ± 12.73	109.6 ± 11.23	0.196
carcass deep (cm)	33.40 ± 6.87	32.80 ± 3.56	29.40 ± 2.96	30.60 ± 2.30	0.442
slaughtering rate (%)	0.499 ± 0.010	0.513 ± 0.005	0.488 ± 0.027	0.492 ± 0.017	0.161

**Table 4:** Effect of dietary concentrate/crude ratio on organ index and non-carcass properties of yak cattle.

Groups	C80	C65	C50	C35	*P*-Value
heart mass/kg	1.072 ± 0.201	0.982 ± 0.172	0.862 ± 0.280	0.974 ± 0.185	0.508
Liver mass/kg	2.306 ± 0.088	2.106 ± 0.142	1.774 ± 0.301	1.924 ± 0.657	0.167
Spleen mass/kg	0.324 ± 0.057	0.312 ± 0.013	0.276 ± 0.076	0.304 ± 0.067	0.631
lung mass/kg	2.010 ± 0.216^b^	2.638 ± 0.435^a^	1.746 ± 0.354^b^	2.000 ± 0.500^b^	0.015
Kidney mass/kg	0.680 ± 0.115	0.594 ± 0.047	0.652 ± 0.268	0.648 ± 0.067	0.840
head mass/kg	8.586 ± 1.279	9.030 ± 0.760	7.300 ± 1.829	7.774 ± 1.516	0.241
hoof quality/kg	3.572 ± 0.430	3.404 ± 0.165	2.950 ± 0.549	3.126 ± 0.500	0.151
skin quality/kg	10.556 ± 1.658	10.550 ± 0.755	8.574 ± 1.901	8.694 ± 1.127	0.062

### Effect of dietary concentrate ratio on yak meat quality

As shown in Table 5, the dietary concentrate ratio had varying effects on the meat quality of different yak muscle groups.In the longissimus dorsi muscle, the pH was significantly higher in the C65 group compared to the C80 group (*P* < 0.05). In LD muscle, the L* (lightness) value was significantly higher in the C65 group than in the C50 group (*P* < 0.05). Drip loss was significantly higher in the C80 group than in the C65 and C35 groups (*P* < 0.05). In the triceps brachii muscle, shear force was significantly higher in the C80 group than in the C35 group (*P* < 0.05). Drip loss was significantly higher in the C80 and C50 groups compared to the C65 group (*P* < 0.05).

**Table 5:** Effect of dietary concentrate/crude ratios on meat quality traits of the longest dorsal muscle, triceps brachii, and biceps femoris of yak (*Bos grunniens*).

position	Meatqualitytraits	Groups				*P*-Value
		C80	C65	C50	C35	
LD	shearing force/N	15.036 ± 1.234	19.672 ± 4.463	16.028 ± 5.072	19.948 ± 4.170	0.163
	pH	5.690 ± 0.198^b^	6.340 ± 0.502^a^	5.922 ± 0.352^ab^	6.000 ± 0.607^ab^	0.034
	L^*^	36.448 ± 3.463^ab^	40.088 ± 5.054^a^	34.672 ± 2.883^b^	37.176 ± 2.823^ab^	0.033
	a^*^	16.502 ± 3.454	16.308 ± 3.442	14.528 ± 3.387	15.488 ± 0.929	0.722
	b^*^	7.332 ± 2.436	7.652 ± 2.550	6.074 ± 1.981	6.792 ± 0.884	0.652
	Drip loss/%	0.084 ± 0.040^a^	0.037 ± 0.018^b^	0.060 ± 0.019^ab^	0.043 ± 0.017^b^	0.047
	Cooking loss/%	0.419 ± 0.025	0.401 ± 0.017	0.418 ± 0.020	0.432 ± 0.024	0.214
AT	shearing force/N	31.894 ± 2.343^a^	25.369 ± 5.404^ab^	27.556 ± 6.269^ab^	24.253 ± 4.081^b^	0.046
	pH	6.230 ± 0.165	5.000 ± 0.418	5.066 ± 1.564	5.088 ± 1.389	0.247
	L^*^	28.384 ± 3.392	32.670 ± 3.440	33.830 ± 2.174	33.648 ± 2.166	0.643
	a^*^	14.476 ± 3.464	13.206 ± 3.907	15.434 ± 2.649	13.482 ± 2.448	0.680
	b^*^	5.862 ± 1.229	5.026 ± 2.183	6.028 ± 1.506	5.622 ± 1.540	0.788
	Drip loss/%	0.123 ± 0.040^a^	0.017 ± 0.006^b^	0.093 ± 0.024^a^	0.049 ± 0.018^ab^	0.032
	Cooking loss/%	0.351 ± 0.050	0.413 ± 0.022	0.400 ± 0.041	0.366 ± 0.065	0.178
BF	shearing force/N	29.926 ± 5.345	29.652 ± 4.493	31.258 ± 5.812	34.271 ± 1.978	0.403
	pH	6.258 ± 0.374^a^	4.640 ± 1.275^b^	5.424 ± 1.351^ab^	4.708 ± 0.982^b^	0.029
	L^*^	44.428 ± 3.935^a^	40.538 ± 3.399^ab^	37.758 ± 4.680^b^	38.654 ± 2.043^b^	0.014
	a^*^	15.200 ± 1.788	15.304 ± 1.651	14.936 ± 1.920	13.368 ± 1.868	0.327
	b^*^	6.736 ± 0.953	7.840 ± 1.405	6.952 ± 1.842	5.904 ± 0.965	0.196
	Drip loss/%	0.081 ± 0.015	0.041 ± 0.017	0.123 ± 0.096	0.055 ± 0.104	0.235
	Cooking loss/%	0.309 ± 0.075^b^	0.424 ± 0.048^ab^	0.317 ± 0.144^b^	0.449 ± 0.048^a^	0.046

LD, Longissimus dorsi muscle; AT, Triceps brachii; BF, Biceps femoris.

In the biceps femoris muscle, the pH was significantly higher in the C80 group compared to the C65 and C35 groups (*P* < 0.05). The L* value was significantly lower in the C50 and C35 groups than in the C80 group (*P* < 0.05). Cooking loss was significantly higher in the C35 group than in the C80 and C50 groups (P < 0.05).

### Analysis of conventional nutrients and micronutrient content of yak longest back muscle by dietary concentrate ratio

As shown in Table 6, the concentrations of conventional nutrients and trace elements in the longissimus dorsi muscle of yaks exhibited varying trends with increasing dietary concentrate proportions, apart from Fe(*P* > 0.10). The iron (Fe) content in the C80 and C35 groups was significantly higher than in the C50 group by 24.12% and 25.68%, respectively (*P* < 0.05). Additionally, the Fe content in the C65 group was 13.08% higher than in the C50 group (*P* < 0.05).

**Table 6:** Regular nutritional components and trace element contents in the longest muscle of Qinghai plateau type yak backs at different ages.

Ltems	Groups				*P*-Value
	C80	C65	C50	C35	
Moisture (g/100g)	75.67 ± 1.90	74.57 ± 0.29	75.73 ± 1.09	74.67 ± 0.50	0.475
Protein (g/100g)	21.07 ± 0.50	21.43 ± 0.81	20.00 ± 2.82	21.93 ± 0.59	0.492
Fat (g/100g)	2.62 ± 1.08	2.31 ± 0.24	2.39 ± 0.81	1.23 ± 0.39	0.158
Ash(g/100g)	1.10 ± 0.10	1.13 ± 0.06	1.20 ± 0.10	1.20 ± 0.00	0.344
Ca(mg/kg)	14.07 ± 0.45	10.84 ± 3.31	12.43 ± 1.20	12.03 ± 0.50	0.252
P(mg/kg)	2063.33 ± 142.95	2220.00 ± 87.18	1760.00 ± 401.49	2223.33 ± 247.05	0.157
Fe(mg/kg)	54.03 ± 1.83^a^	47.17 ± 3.69^ab^	41.00 ± 3.58^b^	55.17 ± 8.19^a^	0.025
Zn(mg/kg)	19.23 ± 3.86	26.83 ± 1.61	22.17 ± 8.24	27.83 ± 8.11	0.342

### Analysis of amino acid and fatty acid content of yak longest back muscle by dietary concentrate ratio

As shown in Table 7, the amino acid composition of the longissimus dorsi muscle in yaks varied with different dietary concentrate-to-roughage ratios.NS differences are shown in Table 7. As shown in Table 8, the fatty acid composition varied among the different dietary concentrate-to-roughage groups. The content of arachidic acid (C10:0) in the C50 group was significantly higher than in the C65 and C80 groups by 69.92% and 63.03%, respectively. NS differences, apart from C10:0, C20:0 and C20:3n6. Similarly, the C35 group exhibited higher arachidic acid levels than the C65 and C80 groups by 65.92% and 58.11%, respectively, with these differences being statistically significant (*P* < 0.05).Regarding arachidonic acid (C20:0), its content in the C35 group was significantly higher than in the C50, C65, and C80 groups by 26.16%, 20.79%, and 29.52%, respectively (*P* < 0.05). Moreover, the content of arachidonic acid (C20:3n6) in the C65 group was significantly lower than in the C50 group (*P* < 0.05) and was 12.80% and 9.33% lower than in the C35 and C80 groups, respectively.

**Table 7:** Amino acid content in the longest dorsal muscle of yaks on diets with refined and coarse ratios (%).

Composition of amino acid	Groups				
	C80	C65	C50	C35	*P*-Value
ASP^#^	1.939 ± 0.027	1.948 ± 0.009	1.924 ± 0.013	1.930 ± 0.009	0.366
GLU^#^	4.959 ± 0.015	4.970 ± 0.012	4.955 ± 0.018	4.960 ± 0.022	0.765
Ser	0.977 ± 0.086	1.153 ± 0.108	1.083 ± 0.126	1.047 ± 0.144	0.385
Gly^#^	1.456 ± 0.203	1.506 ± 0.226	1.511 ± 0.114	1.529 ± 0.097	0.956
His	0.953 ± 0.024	0.937 ± 0.012	0.930 ± 0.011	0.948 ± 0.029	0.537
Arg	1.360 ± 0.009	1.362 ± 0.013	1.358 ± 0.006	1.358 ± 0.009	0.953
The^*^	1.011 ± 0.009	1.025 ± 0.032	1.023 ± 0.015	1.020 ± 0.008	0.812
Ala^#^	1.276 ± 0.022	1.251 ± 0.028	1.258 ± 0.024	1.259 ± 0.018	0.634
Pro 箴	0.866 ± 0.019	0.866 ± 0.007	0.854 ± 0.024	0.867 ± 0.021	0.818
Tyr^#^	0.642 ± 0.018	0.636 ± 0.022	0.627 ± 0.026	0.624 ± 0.012	0.699
Val^*^	1.138 ± 0.015	1.128 ± 0.000	1.161 ± 0.031	1.154 ± 0.030	0.354
Met^*^	0.534 ± 0.029	0.570 ± 0.029	0.557 ± 0.040	0.557 ± 0.017	0.565
Ile^*^	1.106 ± 0.017	1.115 ± 0.012	1.094 ± 0.021	1.107 ± 0.022	0.603
Leu^*^	1.815 ± 0.003	1.805 ± 0.006	1.807 ± 0.005	1.569 ± 0.418	0.446
Phe^*#^	1.010 ± 0.014	1.008 ± 0.015	1.008 ± 0.016	1.015 ± 0.006	0.915
Lys^*^	2.413 ± 0.103	2.349 ± 0.161	2.375 ± 0.164	2.314 ± 0.193	0.888
TRP^*^	0.257 ± 0.021	0.256 ± 0.021	0.294 ± 0.018	0.279 ± 0.021	0.141
TAA	23.713 ± 0.132	23.886 ± 0.050	23.820 ± 0.295	23.776 ± 0.170	0.712

Note: * is an essential amino acid, # is umami amino acid. Different lowercase letters in peer data indicate significant differences between groups (*P* < 0.05).

**Table 8:** Fatty acid content in the longest dorsal muscle of yaks fed diets with refined and crude ratios.

Fatty acid composition	Groups				
	C80	C65	C50	C35	*P*-Value
C4:0	27.01 ± 4.97	28.22 ± 4.96	19.91 ± 6.37	20.61 ± 10.64	0.410
C8:0	12.20 ± 1.29	14.35 ± 1.46	14.21 ± 2.12	14.10 ± 1.78	0.413
C10:0	16.58 ± 2.23^b^	13.49 ± 3.14^b^	44.85 ± 15.89^a^	39.58 ± 11.82^a^	0.011
C12:0	29.01 ± 11.63	25.63 ± 6.27	20.86 ± 10.03	16.60 ± 2.39	0.351
C14:0	50.66 ± 25.72	34.91 ± 4.01	39.43 ± 18.37	48.97 ± 23.24	0.730
C16:0	904.03 ± 445.89	792.32 ± 214.01	992.95 ± 256.11	948.13 ± 323.23	0.884
C16:1n7	139.05 ± 47.07	110.30 ± 46.47	108.03 ± 31.61	113.09 ± 21.43	0.738
C17:0	27.39 ± 7.31	24.59 ± 11.40	39.73 ± 15.74	32.44 ± 13.69	0.502
C18:0	977.30 ± 164.16	1015.05 ± 333.68	1278.51 ± 322.37	1198.52 ± 561.80	0.725
C18:1n9c	1736.55 ± 308.60	1788.96 ± 353.81	2044.44 ± 393.08	1831.93 ± 271.33	0.700
C18:2n6c	732.43 ± 387.92	1081.52 ± 52.73	1058.56 ± 97.19	922.08 ± 96.04	0.226
C18:3n3	46.46 ± 13.42	49.82 ± 2.28	83.39 ± 27.53	76.80 ± 24.90	0.118
C20:0	61.35 ± 7.19^b^	68.94 ± 11.30^b^	64.27 ± 5.94^b^	87.04 ± 5.71^a^	0.016
C20:2	33.47 ± 5.17	38.31 ± 7.73	26.37 ± 22.66	37.82 ± 3.19	0.633
C20:3n6	55.12 ± 5.39^ab^	49.98 ± 14.09^b^	68.55 ± 6.58^a^	57.32 ± 8.20^ab^	0.014
C20:4n6	376.61 ± 81.57	402.29 ± 44.56	359.28 ± 67.98	336.77 ± 89.66	0.736
C20:5n3	67.17 ± 14.36	48.33 ± 14.53	62.75 ± 28.07	67.86 ± 27.15	0.687
C24:0	41.38 ± 17.99	33.78 ± 10.11	42.91 ± 12.60	36.57 ± 5.85	0.793
C22:6n3	54.91 ± 7.47	37.91 ± 7.62	38.64 ± 5.60	38.95 ± 13.17	0.130

## Discussion

### Analysis of dietary concentrate ratio on yak growth performance indexes

With a decrease in the dietary concentrate-to-roughage ratio, there was an increase in the intake of dry matter (DM), organic matter (OM), crude protein (CP), and ether extract (EE), along with a decrease in neutral detergent fiber (NDF) intake. This change was primarily attributed to the higher soluble nutrient content and reduced fiber content, which also contributed to the improved digestibility of DM and OM [8]. In the present study, total weight gain varied significantly with changes in dietary concentrate levels. Yaks fed high-concentrate diets (65–35) exhibited significantly higher total weight gain compared to those fed low-concentrate diets (50–50 and 35–65). This finding aligns with the study by Chen et al.[9], which reported that increasing the dietary concentrate-to-roughage ratio from 30:70–50:50 had a positive effect on the growth performance of housed yaks. Their study also found that yaks fed a low-concentrate diet had higher dry matter intake and average daily gain (ADG) than those fed a high-concentrate diet, which is consistent with the results of the present study.

In this study, increasing the dietary concentrate ratio significantly reduced feed intake, possibly due to the prolonged retention time of concentrate feed in the rumen. The feed-to-weight ratio of yaks in the 65:35 concentrate-to-roughage group was lower than in the other three groups, suggesting that increasing the proportion of concentrate led to a relative reduction in the feed-to-weight ratio. This implies that higher concentrate diets, due to their superior nutritional value, could enhance yak growth performance. Ku et al.[10] similarly found that increasing the dietary concentrate-to-roughage ratio significantly improved total calf weight gain and final slaughter weight, which is consistent with the present findings.

However, in this study, the ADG of yaks in the C65 group was significantly lower than expected. This may be attributed to the physiological adaptations of yaks to high-altitude environments. Yaks have a greater tolerance for roughage, and an excessive increase in dietary concentrate may increase the risk of subacute rumen acidosis. These findings suggest that an optimal concentrate-to-roughage ratio is crucial for maximizing feed efficiency, promoting yak growth, and achieving better fattening outcomes.

### Analysis of dietary concentrate ratio on yak carcass property indexes

Carcass weight, net meat yield, and slaughter rate are key indicators of carcass characteristics in ruminants. These parameters are closely associated with dietary nutrient composition, particularly the concentrate-to-roughage ratio and energy levels. Feeding diets with higher concentrate proportions and energy levels has been shown to enhance slaughter performance in yaks. Studies have demonstrated that increasing the dietary concentrate ratio and energy content improves the growth performance of ruminants, thereby promoting carcass weight gain [11].

Han et al.[12] reported that variations in the proportion of dietary concentrates and roughage significantly influenced slaughter and carcass weights in Red River Yellow cattle, whereas organ indices remained unaffected. Similarly, in the present study, the carcass weight of yaks in the C65 group was significantly higher than that of yaks in the C35 group and C50 group, while no significant differences were observed in organ indices, except for lung mass. Differences in lung mass may be attributed to variations in altitude. Yaks residing at high altitudes (3000–4000 m), where oxygen levels are low, develop larger lungs to increase gas exchange efficiency, thereby maintaining normal metabolism and physiological function despite limited oxygen intake [13].

Mahgoub [14] found that sheep fed high-energy diets exhibited higher slaughter and carcass weights than those fed low- to medium-energy diets. The study also indicated that increasing the concentrate content in the diet improved meat production in Omani sheep by enhancing body weight gain and carcass composition. Similarly, Marino [15] reported that a higher dietary concentrate-to-roughage ratio significantly increased carcass weight, while other slaughter performance metrics remained unaffected. Additionally, Wang et al. [16] found that increasing the concentrate ratio in the diet significantly increased pre-slaughter live weight and carcass weight, whereas non-carcass properties and other carcass traits showed no statistically significant differences.

The results of this study confirm that the dietary concentrate-to-roughage ratio has animpact on yak carcass characteristics. Optimizing this ratio can enhance carcass weight and slaughter rate. However, excessively high or low concentrate levels may not be conducive to improving slaughter performance. Therefore, in practical production settings, dietary concentrate and roughage ratios should be adjusted based on specific conditions to achieve optimal yak slaughter performance indicators.

### Analysis of yak meat quality indexes by dietary concentrate ratio

pH is a critical indicator of meat quality, influencing palatability, tenderness, and cooking properties. It is also closely associated with meat hydration capacity and color [17]. A lower water loss rate and higher cooking yield indicate better water retention, contributing to improved muscle tenderness. Additionally, lower shear force values correspond to increased tenderness and improved meat quality [18]. Meat color is another key quality attribute, with lower brightness (L*) and yellowness (b*) values and higher redness (a*) values generally signifying superior meat color [19].

Alqaisi et al.[20]reported no significant differences in cooking loss, pH, meat color, crude protein (CP), and crude fat (CF) between Holstein calf meat from high- and low-concentrate diets, except for ash content. Similarly, Avilés et al.[21] found no significant difference in the pH of beef from cattle fed high-concentrate diets. In the present study, pH variation was mainly observed in the longissimus dorsi and biceps femoris muscles. The pH of the longissimus dorsi in the C65 group was significantly higher than in the C80 group. This may be attributed to post-slaughter biochemical changes, where muscle metabolism shifts from aerobic to anaerobic respiration, leading to lactic acid accumulation and a subsequent decrease in pH. Additionally, individual yak meat samples with pH values exceeding 5.7 may have been influenced by pre-slaughter stress [22].

Meat color variation was primarily observed in the longissimus dorsi and biceps femoris muscles, with no significant differences in other muscles. This finding aligns with the results of Cuvelier et al. [23], likely due to the similarity in dietary concentrate-to-roughage ratios. Mancini et al.[24] also suggested that lower brightness and yellowness, coupled with higher redness, indicate better meat quality. In the present study, the highest brightness was observed in yaks fed a 65:35 concentrate-to-roughage diet.

Shear force is a crucial measurement of muscle tenderness, an important factor in consumer perception of meat quality. Ozawa et al.[25] noted that higher shear force values indicate reduced tenderness, whereas lower values signify greater tenderness. Drip loss and cooking loss reflect the muscle’s water-holding capacity, with higher retention associated with improved meat quality. Water loss also leads to a reduction in nutrient and flavor retention, negatively impacting texture. Nogalski et al.[26] reported that cooking loss was not affected by diet composition, a finding consistent with the present study. Similarly, SimČiČ et al.[27] compared carcass and meat quality between Cika native bulls and Simmental bulls fed roughage and concentrate, finding that Simmental beef exhibited lower shear force, while cooking and drip loss showed no significant differences.

In the present study, shear force and drip loss rates decreased with an increasing dietary concentrate ratio, corroborating previous findings. This effect is likely due to enhanced intramuscular fat deposition in response to higher dietary energy levels, which improves muscle tenderness and reduces shear force values. Thus, the primary mechanism by which dietary concentrate levels influence meat quality is through increased dietary energy intake, which promotes fat accumulation within the muscle.

### Analysis of conventional nutrients and micronutrient content of yak longest back muscle by dietary concentrate ratio

According to the data, the primary conventional nutrients in beef include protein, fat, moisture, calcium, phosphorus, ash, and cholesterol. Among these, protein is a key determinant of meat quality, while fat content influences tenderness and nutritional value. Additionally, ash content serves as an important indicator of mineral accumulation in meat [28,29]. Water distribution within the carcass is uneven, with muscle containing approximately 70% to 80% water. In addition to water, yak meat is rich in proteins found within muscle tissue, and its mineral content is approximately 1.5% [30].

Marino et al.[15] reported no significant differences in the conventional nutrient composition or micronutrient content of young Bodorian calves when the dietary concentrate level was increased. In the present study, variations in iron (Fe) content among trace elements were observed, which may be attributed to differences in feed composition and individual difference. Similarly, Luana França dos Anjos [31] found that high-concentrate diets in sheep increased dry matter intake, leading to higher fat and protein content in the meat.

In this study, as the dietary concentrate ratio increased, fat content exhibited an upward trend, while protein content first increased and then decreased. However, these trends were not statistically significant, likely due to the relatively small variations in concentrate-to-roughage ratios among the diet groups. High-protein diets have been shown to enhance carcass protein content and promote fat deposition. Adiwimarta et al.[32] also demonstrated that high-protein feeds improve rumen conditions by increasing the consumption of dry matter (DM), crude protein (CP), crude fiber (CF), and nitrogen-free extracts (NFE), ultimately exerting a positive effect on production performance.

### Analysis of amino acid and fatty acid content of the longest muscle of yak back by dietary concentrate ratio

Protein content plays a crucial role in determining the nutritional value of muscle. Since proteins are composed of amino acids, a higher and more diverse amino acid profile is indicative of superior meat quality [33]. Amino acids that contribute to the development of meat flavor are referred to as flavor amino acids, including arginine, glutamic acid, glycine, and aspartic acid, with glutamic acid playing a dominant role [34]. In the present study, all experimental groups contained 17 amino acid species, with total amino acid percentages of 23.713% in the C80 group, 23.886% in the C65 group, 23.820% in the C50 group, and 23.776% in the C35 group. No significant differences were observed among the groups. Rousset-Akrim et al.[35] identified aspartic acid, glutamic acid, serine, glycine, isoleucine, phenylalanine, and proline as key precursors of meat flavor, with their levels increasing as leanness improved. Aspartic acid is known for its role in preventing heart disease, enhancing liver function, regulating blood pressure, and alleviating muscle fatigue. Arginine, a semi-essential amino acid, promotes wound healing and boosts immune function. Glutamic acid has therapeutic effects on peptic ulcers, aids in trauma recovery, and contributes to ammonia detoxification by forming glutamine [36]. In yak longissimus dorsi muscle, glutamic acid was the most abundant amino acid, with its content reaching 4.97% in the C65 group. Among all flavor-related amino acids, glutamic acid exhibited the strongest umami taste, and yak meat contained higher levels of glutamic acid compared to other flavor amino acids.

The composition and concentration of various fatty acids in livestock and poultry meat directly influence meat quality. In ruminants, linoleic acid and linolenic acid undergo biohydrogenation in the rumen, forming conjugated linoleic acid, which has demonstrated health benefits, including anticancer properties, cardiovascular disease prevention, and immune function enhancement [37].

In this study, the levels of C12:0, C16:1n7, and C20:4n6 increased with a higher dietary concentrate ratio, whereas the contents of C10:0, C18:1n9c, C20:0, and C20:3n6 showed a decreasing trend. These findings align with those of Jeon [38], who reported that feeding silage mulberry leaves significantly increased the unsaturated fatty acid content in the longissimus dorsi muscle of cattle. However, differences in fatty acid composition between Jeon’s study and the present experiment may be attributed to factors such as breed variations, feeding systems, rumen microbiota composition, diet formulations, and feeding management practices.

## Conclusion

Appropriately increasing the ratio of protein to non-protein in the feed is beneficial for the growth performance, carcass characteristics and meat quality of raised yaks.Among the tested concentrate-to-roughage ratios of 80:20, 50:50, and 35:65, a 65:35 ratio was found to be the most effective for Qinghai Plateau-type yaks. This ratio led to higher average daily and total weight gain, improved body slant length growth, and enhanced carcass traits and meat quality, ultimately optimizing production performance. However, excessively high concentrate levels did not yield additional benefits. Therefore, under the conditions of this study, a 65:35 concentrate-to-roughage ratio was determined to be the optimal dietary composition for Qinghai Plateau-type yaks.
